# Incidence of Neonatal Mortality and the Factors Influencing Neonate Mortality in Neonatal Intensive Care Unit in Northern Ethiopia: A Prospective Cohort Study

**DOI:** 10.4314/ejhs.v35i1.2

**Published:** 2025-01

**Authors:** Gebrhud Berihu Haile, Tensay Kahsay Weldegebreal, Desta Hailu Aregawi, Daniel Birhane Berhe, Tomas Amare Abraha

**Affiliations:** 1 Mekelle University, Pediatrics and Child Health Nursing, Mekelle, Ethiopia; 2 Mekelle University, School of Nursing, Mekelle, Ethiopia; 3 Mekelle University, Department of Midwifery, Mekelle, Ethiopia

**Keywords:** Incidence, Predictors, Neonates, Neonatal Mortality, Northern Ethiopia

## Abstract

**Background:**

Neonatal mortality remains high globally, with an estimated 2.4 million neonatal deaths in 2020. In Ethiopia, neonatal mortality is particularly concerning. Despite some research, data on neonatal mortality incidence and influencing factors are limited. This study aimed to assess the incidence of neonatal mortality and identify factors affecting neonatal mortality in intensive care units.

**Methods:**

A prospective follow up cohort study was conducted in public hospitals in northern Ethiopia from October 2021 to May 2022, involving 334 neonates admitted to the intensive care unit. Systematic random sampling was employed to select participants, and data were collected through structured interviews and chart reviews. Data were analyzed using STATA 14, with a Cox model to calculate hazard ratios (AHR) and 95% confidence intervals (CI), considering statistical significance at P<0.05.

**Results:**

The neonatal mortality incidence rate was 23.21 per 1000 person-days (95% CI: 17.15, 31.41). Significant predictors of neonatal mortality included: no antenatal care (ANC) utilization (AHR: 3.69; 95% CI: 1.62, 8.42), a 5-minute APGAR score <7 (AHR: 0.38; 95% CI: 0.19, 0.77), prematurity (AHR: 0.34; 95% CI: 0.13, 0.90), and perinatal asphyxia (PNA) (AHR: 0.17; 95% CI: 0.04, 0.66).

**Conclusion:**

The neonatal mortality rate was high. ANC utilization, 5-minute APGAR score <7, prematurity, and PNA were significant predictors. Healthcare professionals should prioritize these factors to improve neonatal survival in intensive care units.

## Introduction

Incidence of neonatal mortality is refers the probability of neonatal deaths within the first 28 days of life, measured over a specific area and period (1). The neonatal period is characterized by rapid physiological changes, with key development processes, such as feeding and bonding, taking place during this time(2,3). In 2020, approximately 2.4 million newborns died globally, with around 7,000 deaths occurring daily. While the global neonatal mortality rate (NMR) is declining, significant disparities remain, especially in regions like South Asia and Sub-Saharan Africa, where neonatal mortality rates are among the highest (4,5).

In Ethiopia, the neonatal mortality rate was 30 per 1,000 live births in 2019, according to the Ethiopian Mini Demographic and Health Survey. Despite declines in under-five and overall childhood mortality, neonatal mortality reductions have been less pronounced(6). By 2030, the Sustainable Development Goal (SDG) target is to reduce the NMR to 12 per 1,000 live births(7,8).

Malnutrition, infections, and neonatal conditions contribute to over two-thirds of childhood deaths in Ethiopia. Factors such as low birth weight, prematurity, infections, asphyxia, and birth trauma account for nearly 80% of neonatal deaths(9,10). Studies from other parts of Ethiopia highlight sepsis, perinatal asphyxia, prematurity, respiratory distress syndrome (RDS), and low birth weight as major contributors to neonatal mortality in intensive care units (NICUs) (10,11,12). Improving birth care practices is crucial for reducing neonatal mortality, as recommended by the World Health Organization (WHO). Essential newborn care (ENC) practices, such as clean cord care, thermal regulation, and breastfeeding initiation within the first hour, play a vital role (9). The Ethiopian government has implemented evidence-based interventions through USAID initiatives to improve neonatal care at various levels(13). However, there is limited data regarding the incidence and factors influencing neonatal mortality in Ethiopia's NICUs. This study aims to fill this gap by assessing these factors in Mekelle City's public hospitals.

## Methods

**Study setting, design, and period**: This study was conducted in public hospitals in Mekelle city, located in the Tigray region of Ethiopia. These hospitals include referral, general, and primary care facilities. Mekelle is approximately 783 km from Addis Ababa, with a population of around 611,574 residents in 2024(14). An institution-based prospective cohort study was conducted from October 23, 2021 to May 23, 2022.

**Study population**: The study population consisted of all neonates admitted to the NICU of selected public hospitals in Mekelle city.

**Inclusion and exclusion criteria**: Neonates aged 28 days or younger admitted to the NICU during the study period were included. Neonates referred from other hospitals were excluded to avoid double-counting.

**Sample size determination**: A Cox proportional hazard model was used to calculate the sample size, assuming a type I error of 5%, power of 80%, and a neonatal death probability of 16.5%(11). A final sample size of 334 was determined after accounting for a 10% non-response rate.

**Sampling technique**: Three out of five governmental public hospitals in Mekelle city were randomly selected using a simple random sampling technique. Study participants were selected through systematic random sampling from each hospital.

**Data collection**: Data were collected using a structured checklist adapted from WHO standard questionnaires(15). Data collectors, including five midwives and two MSc supervisors, conducted structured interviews and chart reviews. A pretest was conducted at Axum Referral Hospital to ensure clarity and consistency in the data collection process.

**Data processing and analysis**: Data were entered using EpiData v4.4 and analyzed using STATA 14 and SPSS 20. Descriptive statistics were used to summarize the data, and Kaplan-Meier and life tables estimated the survival function. Cox proportional hazards regression was used to identify predictors of neonatal mortality, with statistical significance set at P<0.05.

**Ethics**: Ethical approval was granted from the Institutional Review Board of Mekelle University's College of Health Sciences (ERC 1523/2020). Informed consent was obtained from all participants, and confidentiality was maintained throughout the study.

## Results

**Socio-demographic characteristics**: A total of 334 neonates participated in the study. Most mothers (62.3%) resided in the city, with 89.8% married. The majority (96.1%) of them was Orthodox Christians, and 53.3% were housewives (Table 1).

**Table 1:** The socio-demographic participants for assessment of neonatal mortality incidence in the intensive care unit of public hospitals, Tigray, Ethiopia, 2022

Variables		Frequency(%)
Age at first marriage	<18	146 (43.7)
	≥18	188 (56.3)
Age of mother at first birth in years	<20	162 (48.5)
	20-34	168 (50.3)
	>34	4 (1.2)
Mother's current age	15-19	22(6.6)
	20-24	87(26.0)
	25-29	118(35.3)
	30-34	59(17.7)
	35-39	35(10.5)
	≥40	13(3.9)
Place of residence	Rural	126(37.7)
	Urban	208(62.3)
Marriage Status	Married	300(89.8)
	Unmarried	26(7.8)
	Others***	8(2.4)
Religion Status	Orthodox	321(96.1)
	Muslim	10(3.0)
	Others **	3(.9)
Occupation	house wife	178(53.3)
	Private	63(18.9)
	Governmental	69(20.7)
	Student	24(7.2)
Educational status of mother	Unable to read and write*	44(13.2)
	Primary school	83(24.9)
	Secondary school	110(32.9)
	College & above	97(29.0)
Family income in ETB	Less than 500	19(5.7)
	500-1500	136(40.7)
	More than 1500	179(53.6)
From home to nearest health institution distance	≤5km	252(75.4)
	5-10km	59(17.7)
	More than 10km	23(6.9)

ETB: Ethiopian birr, Others *** widowed and divorced, ** protestant and catholic, *unable to read and write and able to read and write=unable to read and write

**Maternal and obstetric characteristics**: Among mothers, 43.7% married before the age of 18, and 35.3% were aged 25-29 at the time of the study. Most neonates (60.8%) were born at 37-42 weeks of gestation, and 66.5% were breastfed within the first hour (Table 2).

**Table 2:** Maternal and obstetric related characteristics of neonates admitted to intensive care unit in Mekelle city, selected public hospitals, Tigray, Ethiopia, 2022

Variables		Frequency(%)
Gravidity	one	109(32.6)
	two	84(25.1)
	≥three	141(42.2)
Parity	One	135(40.4)
	Two	77(23.1)
	three and above	122(36.5)
Previous abortion history	Yes	92(27.5)
	No	242(72.5)
Complications with present pregnancy and delivery	yes	293(87.7)
	No	41(12.3)
If yes, type of complications;		
APH	yes	37(11.1)
	No	297(88.9)
PPH	yes	24(7.2)
	No	310(92.8)
PIH	yes	48(14.4)
	No	286(85.6)
Prolonged labor	Yes	81(24.3)
	No	253(75.7)
PROM	yes	182(54.5)
	no	152(45.5)
NRFHRP	yes	86(25.7)
	no	248(74.3)
Other *	yes	91(27.2)
	no	243(72.8)
Known medical disease during current pregnancy If yes, type of medical disease(n=165)	yes	168(50.3)
	no	166(49.7)
	HIV/AIDS	25(7.5)
	DM	23(6.9)
	UTI	21(6.3)
	Anemia	56(16.8)
	Others**	43(12.9)
Utilization of ANC	yes	307(91.9)
	no	27(8.1)
If yes, number of visit	<4 visit	84(25.1)
	≥4 visit	223(66.8)
Labor onset	spontaneous induced	256(76.6)
		78(23.4)
Rupture of membrane time (ROM)	<12 hours	221(66.2)
	≥12 hours	113(33.8)

Others, **Hepatitis, CHF, hypertension, TB, Asthma, and *precipitated labor, post term, obstructed labor, Rh incompatible, IUGR, polyhyramious and oligohydramious etc.

**Neonatal Characteristics**: The study included 210 male neonates (62.9%). Approximately 65.3% were ≤1 day old at admission, and 54.19% had an APGAR score of ≥7 at 1 minute (Table 3).

**Table 3:** Neonatal related characteristics for assessment of neonatal mortality incidence in intensive care unit of public hospitals, Tigray, Ethiopia, 2022

Variables		Frequency(%)
Sex	male	210(62.9)
	female	124(37.1)
During admission age of neonate	≤ 1 day	218(65.3)
	1-7 days	97(29.0)
	>7 days	19(5.7)
GA during birth time	≤36 weeks	115(34.4)
	37-42 weeks	203(60.8)
	>42 weeks	16(4.8)
Breastfeeding initiation within one hour	yes	212(63.47
	No	100(29.94
	unknown	22(6.59)
APGAR score at 1^st^ minute	<7	118(35.33)
	≥7	181(54.19
	unknown	35(10.48)
APGAR score at 5^th^ minute	<7	120(35.93)
	≥7	179(53.59)
	unknown	35(10.48)
Neonate referral status	inborn	210(62.87)
	outborn	124(37.13)
Birth injury	yes	102(30.54)
	No	232(69.46)
Resuscitation status of neonate	yes	113(33.8)
	No	221(66.2)
Does a neonate received any treatment in NICU?	yes	319(95.5)
	no	15(4.5)
If yes, type of treatment; Antibiotics	yes	284(85.0)
	No	50(15.0)
Fluid	yes	293(87.7)
	No	41(12.3)
Oxygen	yes	194(58.1)
	No	140(41.9)
Surgery	Yes	34(10.2)
	No	300(89.8)
At the end of follow up results of neonate	Discharge	191(57.3)
	Transfer	59(17.8)
	Death	42(12.6)
	Others*	41(12.3)
Neonatal Outcome	Censored	292(87.4)
	Death	42(12.6)

Others, ** spinal cord injury, cranial nerve injury, peripheral nerve injury, and * alive at the end of follow up time, left against medical advice, escaped

**Kaplan-Meier analysis**: Kaplan-Meier estimates revealed that neonates had a high probability of survival in the initial days, with the likelihood decreasing as follow-up time increased (Figure 1).

**Incidence of neonatal mortality**: The incidence rate of neonatal mortality was 23.21 per 1,000 person-days (95% CI: 17.15, 31.41). Of the neonates, 12.6% died during the study period, resulting in a neonatal mortality rate of 126 per 1,000 live births. The majority of deaths occurred within the first 7 days of admission (Table 4).

**Table 4:** Life table analysis for incidence neonatal mortality in intensive care unit of public hospitals, Tigray, Ethiopia, 2022

Interval time in days	Total No at start	Death	Censored	ENNA	PD %	PS %	CPS %	95% CI
0-7	334	32	200	229	13.7	86.3	86.1	81.82, 90.91
7-14	102	6	66	68.5	9.8	90.2	78.6	70.77, 84.89
14-21	30	2	19	20.5	10.3	89.7	70.9	57.26, 81.21
21-28	9	2	7	5.5	37.7	62.5	45.1	16.40, 70.65

Censored=neonates either of discharge, transferred, left against medical advice, escaped and alive at the end of follow up time; CPS=Cumulative probability of surviving at the end of 7, 14, 21 and 28 days, ENNA=effective number of neonates at risk during interval time, PD=probability of dying during interval time, PS=probability of surviving during interval time.

**Bivariate and multivariable analysis**: Bivariate analysis identified several predictors of neonatal mortality. In multivariable analysis, ANC utilization, prematurity, 5-minute APGAR score <7, and perinatal asphyxia were found to significantly increase the risk of neonatal mortality. Specifically, neonates whose mothers had no ANC visits were 3.69 times more likely to die. Neonates with a 5-minute APGAR score <7 had a 62% higher risk of death, while premature neonates faced an 83% higher risk. Perinatal asphyxia was associated with a 66% higher risk of mortality.

Overall, the study highlights a high neonatal mortality rate in Mekelle's NICUs, with ANC utilization, prematurity, 5-minute APGAR score <7, and perinatal asphyxia identified as key predictors. Healthcare providers should prioritize these factors to enhance neonatal survival outcomes (Table 5).

**Table 5:** Bivariate and multivariable Cox regression analysis for for incidence neonatal mortality in intensive care unit of public hospitals, Tigray, Ethiopia, 2022

Variables		Survival status		CHR (95% CI)	AHR(95% CI)

		Death	Censored		
ANC utilizations	No	9	18	5.18(2.42, 11.6)	3.69(1.62, 8.42)*
	yes	33	274	1	1
Source of referral	Out born	26	108	.89(.47, 1.67)	2.04(.87, 4.75)
	Inborn	16	184	1	1
Membrane rapture time	≥12hours	32	81	6.19(3.4,12.62)	1.9(.82, 4.68)
	<12hours	10	211	1	1
Sepsis	Yes	16	178	3.15(1.68, 5.92)	2.40 (.93, 6.18)
	No	26	114	1	1
Resuscitated neonate	yes	10	83	1.22(.601, 2.49)	1.7(.70, 4.54)
	No	32	209	1	1
APGAR score at first minute	<7	33	85	.32(0.15, 0.55)	.72(.36, 1.44)
	≥7	6	175	1	1
	unknown	3	32	1.8(.70,3.09)	1.2(.24, 6.54)
MAS	yes	13	74	.73(.38, 1.42)	.52(.21, 1.25)
	No	29	218	1	1
APGAR score at fifth minute	<7	35	85	.18(.09, 0.36)	0.38(0.19, 0.77)*
	≥7	4	175	1	1
	unknown	3	32	.99(.28, 3.52)	0.39(0.12, 1.32)
Birth trauma	yes	8	94	1.78(.82, 3.85)	.74(.28, 1.97)
	No	34	198	1	.1
Initiation of BF within 1hr	yes	5	207	1	1
	no	33	67	2.59(1.73, 3.87)	1.61(.82 3.16)
	Unknown	4	18	1.1(1.61,5.70)	1.37(0.64, 4.03)
prematurity	yes	35	79	.12(.05, 0.27)	0.34(.13, 0.90)*
	no	7	213	1	1
Complication during birth	yes	40	253	.35(0.08, 1.48)	1.74(.36 8.31)
	no	2	39	1	1
PNA	yes	39	115	.08(.02, 0.26)	0.17(.04, 0.66)*
	no	3	177	1	1

* Significant at (p-value ≤ 0.05)

## Discussion

The aim of this study was to assess the incidence of neonatal mortality and the factors influencing it in the Neonatal Intensive Care Unit (NICU). The findings indicate an incidence rate of 23.21 neonatal deaths per 1000 person-days (95% CI: 17.15, 31.41). Multivariable analysis identified several key predictors of neonatal mortality in the NICU, including antenatal care (ANC) utilization, a fifth-minute APGAR score below seven, prematurity, and neonates diagnosed with perinatal asphyxia (PNA). These results highlight the significant challenges faced by neonates in intensive care.

During the study period, the overall neonatal mortality rate was 12.6% (95% CI: 9.01, 16.14), which translates to 126 deaths per 1000 live births. This is significantly higher than the national neonatal mortality rate (NMR) of 30 per 1000 live births reported in the 2019 Ethiopia Mini Demographic and Health Survey (EMDHS)(6). This discrepancy can be explained by the fact that NICU admissions are typically reserved for neonates facing severe health challenges such as extreme prematurity, low birth weight, congenital abnormalities, infections, or complications during delivery. These high-risk neonates are more susceptible to death compared to the average newborn. In contrast, the national NMR includes both healthy full-term newborns and those with complications, thus lowering the overall mortality rate. Since NICUs primarily treat critically ill infants, the case fatality rate in these units tends to be higher.

The incidence rate of neonatal mortality in this study (23.21 per 1000 person-days) aligns with findings from studies at Wolaita Sodo University and Arba Minch University, where rates were 27 and 31.6 deaths per 1000 person-days, respectively(10,11). However, this rate is lower than those reported in other Ethiopian regions, such as Gurage and Amhara, where the rates were 36.9 and 53.0 per 1000 person-days(18,19). The variations in incidence rates across different regions may reflect differences in healthcare infrastructure, resource availability, and regional variations in maternal and neonatal health practices.

The median survival time for neonates in this study was 24 days. Similar to other studies conducted at various universities in Ethiopia(10,11,20) and international research in countries like Ghana, Kenya, and Iraq (21,22,23) the majority of neonatal deaths occurred within the first 24 hours (35.71%) and during the next seven days (40.48%). This early mortality suggests that complications arising from pregnancy, labor, or delivery that are not timely recognized and managed can lead to rapid deterioration. Low birth weight and prematurity were identified as major risk factors for early mortality, which could be exacerbated by the current NICU's capacity to manage such conditions effectively.

The study found that neonates born to mothers who did not attend ANC were 3.69 times more likely to die compared to those whose mothers received adequate ANC. This is consistent with other studies across Ethiopia (11,12,24,25) and emphasizes the critical role of ANC in preventing neonatal mortality. ANC visits allow for early detection of risks such as infections or preterm labor, management of complications, and better birth outcomes through skilled birth attendance. Adequate ANC ensures that potential health issues are addressed early, promoting both maternal and neonatal health.

Furthermore, neonates with an APGAR score below seven at five minutes were 62% more likely to die compared to those with a higher score. This finding corroborates previous studies from Ethiopia, Cameroon, and the Democratic Republic of Congo(10,12,25,26,27,28). A low APGAR score at five minutes is indicative of ongoing physiological distress, particularly in relation to breathing, heart rate, and neurological stability. Such scores are associated with an increased risk of infections, brain damage, hypoxia, and organ failure, all of which contribute to higher mortality rates. Early and effective resuscitation can help mitigate some of these risks, but a persistently low score signals a need for urgent medical intervention to prevent further complications.

The study also revealed that neonates diagnosed with PNA had a 66% higher likelihood of death compared to those without this diagnosis, this finding is consistent with other studies in Ethiopia(11,18,19). PNA is a leading cause of neonatal mortality due to its severe impact on oxygen supply during birth and the subsequent risk of neurological and organ damage. Prompt and effective neonatal care is essential to improve survival rates for neonates with PNA, as early detection and resuscitation can prevent long-term damage and death.

Prematurity was another significant factor, with preterm neonates being 83% more likely to die compared to term neonates. This finding aligns with research conducted locally and globally (23,28,29,30), showing that premature infants face multiple health challenges, including underdeveloped lungs, the risk of infections, and other life-threatening conditions. Without timely interventions such as respiratory support and appropriate nutrition, preterm neonates are at a higher risk of mortality.

The limitations of the study include the potential overestimation of median survival times due to a high level of censorship in the analysis. Neonates who were discharged early or transferred to another facility before reaching 28 days were assumed to have survived until the study's conclusion, which may have impacted the accuracy of survival estimates.

In conclusion, this study found a high incidence of neonatal mortality in the NICU, with a median survival time of 24 days. The main predictors of neonatal mortality included low APGAR scores at five minutes, lack of ANC utilization, prematurity, and PNA. Healthcare providers should focus on improving care for neonates with low APGAR scores, those born to mothers without ANC follow-up, premature neonates, and those with PNA. Additionally, healthcare professionals should be vigilant during the early neonatal period, as this is when mortality is most prevalent. Future studies should include follow-up assessments of neonates after discharge to better understand survival trends beyond the NICU.

## Figures and Tables

**Figure 2:**
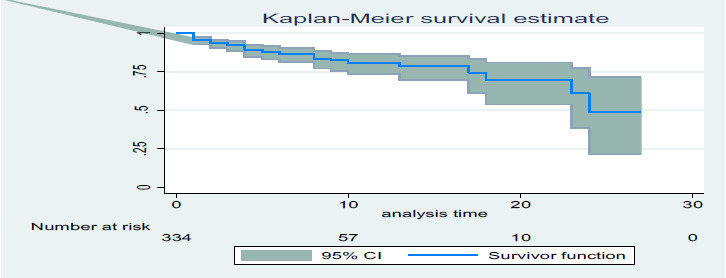
Kaplan-Meier survival estimate of neonates in NICU of Mekelle city, selected public hospitals, Tigray, Ethiopia, 2022
